# The Western Lancet

**Published:** 1844-09

**Authors:** 


THE WESTERN LANCET.

We received the Western Lancet, for July, after the matter for our present number had been composed, and just as the last sheets were going to the press. It contains a personal attack upon us, which the author himself, Dr. Peter, seems to regard as rather 'uncourteous.' We have no space left us, at present, if we had the disposition, to embark in a personal quarrel with that gentleman. But we must tell him, that he has been miserably hoaxed, if he has credited any story of our 'watching stages and steamboats, and *besetting* students of medicine.' True, we have occasionally met with students on their way to Lexington, and, as the subject naturally came up, have not hesitated to tell them what we knew about the means of instruction, and the character of some of the professors there. If Dr. P., and some of his colleagues have not made quite as free with the Faculty of the Medical Institute, both in public taverns, and in their lectures, their pupils have done them great injustice. For ourselves, we have been obliged, on more than one occasion, in self-defence, both to write and speak with some plainness of Dr. Peter and one of his colleagues, and it seems that we shall have that unpleasant duty again to discharge in relation to the former. At present, all we have room to say is, that his accusation of 'watching stages and steamboats for students, and besetting them with *plausible inducements*,' so far as it implicates us, is false. Y.

